# Plague in China 2014—All sporadic case report of pneumonic plague

**DOI:** 10.1186/s12879-016-1403-8

**Published:** 2016-02-19

**Authors:** Yun-fang Li, De-biao Li, Hong-sheng Shao, Hong-jun Li, Yue-dong Han

**Affiliations:** Radiology Department, Beijing YouAn Hospital, Capital Medical University, Beijing, 100069 China; Radiology Department, the First People’s Hospital of Yumen, Gansu, China; Imaging Diagnostic Center, Lanzhou General Hospital, Lanzhou Command, PLA, Lanzhou, 730050 China; Department of Interventional Radiology, Rehabilitation Center Hospital, of Gansu Province, Gansu, China

**Keywords:** Plague, Clinical feature, Diagnosis, Treatment, Quarantine

## Abstract

**Background:**

*Yersinia pestis* is the pathogen of the plague and caused three pandemics worldwide. Pneumonic plague is rarer than bubonic and septicemic plague. We report detailed clinical and pathogenic data for all the three sporadic cases of pneumonic plagues in China in 2014.

**Case presentation:**

All the three patients are herders in Gansu province of China. They were all infected by *Yersinia pestis* and displayed in the form of pneumonic plague respectively without related. We tested patient specimens from the upper (nasopharyngeal swabs) or the lower (sputum) respiratory tract and whole blood, plasma, and serum specimens for *Yersinia pestis*. All patients had fever, cough and dyspnea, and for patient 2 and 3, unconscious. Respiratory symptoms were predominant with acute respiratory failure. The chest X-ray showed signs consistent with necrotizing inflammation with multiple lobar involvements. Despite emergency treatment, all patients died of refractory multiple organ failure within 24 h after admission to hospital. All the contacts were quarantined immediately and there were no secondary cases.

**Conclusions:**

Nowadays, the plague is epidemic in animals and can infect people who contact with the infected animals which may cause an epidemic in human. We think dogs maybe an intermediate vector for plague and as a source of risk for humans who are exposed to pet animals or who work professionally with canines. If a patient has been exposed to a risk factor and has fever and dyspnea, plague should be considered. People who had contact with a confirmed case should be isolated and investigated for F1 antigen analysis and receive post-exposure preventive treatment. A vaccination strategy might be useful for individuals who are occupationally exposed in areas where endemically infected reservoirs of plague-infected small mammals co-exist.

## Background

Plague caused by bacterium *Yersinia pestis* (*Y. pestis*) that belongs Yersiniavan Loghem, is a vector-borne disease which caused millions of human deaths in the Middle Ages [1]. Nowadays, human plague shows a relatively low incidence but a high mortality [2]. It is a rapidly progressing, highly infectious, and highly feared disease that is likely to be fatal without prompt antibiotic treatment [3]. Currently, the disease is still endemic in many areas of the world presenting a serious threat to humans in Asia, America, and Africa [4]. Approximately 1000 cases of plague are reported by the World Health Organization in 2013 worldwide [5]. According to the China CDC [6], there is no widespread plague in China in the past five years, but only some sporadic cases have happened. Six plague patients were reported in 2010, with two deaths; one each death case was reported respectively in 2011 and in 2012; no case was reported in 2013 in China. As in 2014, there are three sporadic cases of plague in China. We report detailed clinical and pathogenic information for the three cases. Human infection with *Y. pestis* usually manifests itself in three forms depending on the route of infection: bubonic, septicemic and pneumonic. The majority of cases are bubonic or septicemic [7]. Bubonic and septicemic infections, usually caused by the bites of infected fleas that live on or near the mammalian host reservoirs. Cases of pneumonic infection have always been much rarer, even during large outbreaks in the past, but our three cases are all primary pneumonic plague.

## Case presentation

We report data for three sporadic patients who were respectively admitted to hospital in July, September, and October, 2014, in Gansu province of China. Their medical records were compiled and reviewed by their attending physicians.

*Y. pestis* was isolated from specimens from the upper (nasopharyngeal swabs) or the lower (sputum) respiratory tract, whole blood, plasma, and serum specimens. The blood, sputum, and nasopharyngeal swab specimens were collected for strain isolation and for serological tests to determine the F1 antibody concentration via indirect hemagglutination assay (IHA) [8, 9]. We also used reverse IHA to detect the *Y. pestis* F1 antigen from sputum and throat samples [10].

### Demography and epidemiology of the patients

Patient 1 was a 38-year-old man has no underlying disease, who developed symptoms of fever two days after exposure to a herding dog that had seized a marmot on July 11, 2014. High fever and arrhythmia showed 2 day after the onset of illness. He lived in Yumen City, Gansu, and was admitted to the Yumen People’s Hospital on July 13.

Patient 2, a 46-year-old man, has no underlying conditions, who presented to the Yumen People’s Hospital with symptoms of high fever, cough and unconscious on Oct. 1, 2014. This patient was a shepherd who worked at Subei County. He had unknown exposure before the onset of symptoms. He lived in Yumen City, Gansu.

Patient 3 was a 50-year-old man who was a herder and lived in Subei County, Yumen City, Gansu. He had no underlying disease, and unknown exposure history before the onset of symptoms. Patient 3 had high fever, cough and dyspnea two hours before admission to Subei Hospital on Oct. 14, 2014. He was unconscious when he presented to the hospital.

The demographic and epidemiologic characteristics of the three patients are summarized in Table 1.

**Table 1:** Demographic, epidemiologic, clinical features, complications, treatment, and clinical outcomes of three patients infected with plague^a^

Characteristic	Patient 1	Patient 2	Patient 3
Age (yr.)	38	46	50
Sex	Male	Male	Male
Occupation	Herder, farmer	Herder	Herder
Underlying conditions	None	None	None
Area of origin	Gansu, China	Gansu, China	Gansu, China
Date of illness onset	July 13, 2014	September 29, 2014	October 2, 2014
Date of admission	July 15, 2014	October 1, 2014	October 14, 2014
Vital Sign			
Temperature (°C)	37.5; 39.5	38.5	40.7
Pulse (b/min)	130; 150	158	144
Respiratory (b/min)	21; 33	78	37
Blood pressure (mmHg)	83/36; 77/43	40/20	100/60
Spo2	77 %; 82 %	-^a^; 77 %	72 %
Clinical features			-
Unconscious	No	Yes	Yes
Fatigue	Yes	-	-
Cough	Yes	Yes	Yes
Sputum	Yes^b^	-	-
Haemoptysis	No	-	-
Dyspnea	Yes	Yes	Yes
Chest pain	Yes	-	-
Abdominal pain	No	-	-
Nausea or vomiting	Yes	-	No
Myalgia	Yes	-	-
Haemorrhage spots	No; Yes	Yes	No
Skin rash	No	No	No
Complications			
Septic shock	Yes	Yes	
ARDS	Yes	Yes	Yes
Acute renal damage	Yes	Yes	
Acute liver injury	Yes	Yes	
Encephalopathy	Yes	Yes	
DIC	Yes	Yes	
Electrolyte disturbance	Yes	Yes	
Oxygen therapy	Mask	Mask	Mask
Antibiotic therapy	Cefoperazone, sulbactam streptomycin, gentamicin	No	Clindamycin
Length of stay in hospital	14 hours	3 hours	1 · 3 hour
Date of death	July 16, 2014	October 1, 2014	October 14, 2014

Results for when the patients presented and the patients’ most abnormal result during disease progression are given. If the reading at presentation was the most abnormal reading, only one result is given

^a^:“-”cannot measure/cannot inquiry

^b^Pink frothy sputum

### Determination of causative pathogens

We confirmed that all the three patients were infected with *Y. pestis* by means of real-time RT-PCR, microscopy, and IHA. The IHA titer of antibody to F1 antigen of patient 1 was 1:40 (serum); and the reverse IHA antibody titres were 1:6400 (nasopharyngeal swab specimen) and 1:12800 (sputum). PCR tests for *Y. pestis* fra and pla showed positive results [11, 12]. On July 18, *Y. pestis* strains were isolated from sputum, blood and nasopharyngeal swab samples and identified through bacteriophage lysis test and PCR.

The IHA titer of antibody to F1 antigen of patient 2 was 1:160 (lymphatic fluid) and 1:2560 (serum); the reverse IHA titre was 1:1024 (serum). PCR test for *Y. pestis* fra and pla was positive [11, 12]. On October 3, *Y. pestis* strains were isolated from sputum, lymphatic fluid and blood samples, Gram-negative curtobacterium with two obtuse and trachychromatic ends was found by thick smear microscopy, and the bacteriophage lysis test was positive.

Patient 3 Gram-negative curtobacterium with obtuse and trachychromatic on both ends was found by thick smear microscopy from sputum and blood samples, antibody to F1 antigen was detected at a titre of 1:800 through IHA (serum); the reverse IHA showed titres of 1:5120 (serum). PCR test for *Y. pestis* fra and pla was positive [9, 10], and identified by bacteriophage lysis test, microscopy and PCR.

All the pathogen results of the patients are also shown in Table 2.

**Table 2:** Laboratory measurements in three patients with plague infection

	Normal range	Patient 1	Patient 2	Patient 3
Haemoglobin (g/L)	110–165	206	201	
Total white cells (×10^9^ cells/L)	3 · 5–10 · 0	8 · 9	58	
Neutrophils (×10^9^ cells/L)	1 · 2–6 · 8	5 · 79	41 · 8	
Lymphocytes (×10^9^ cells/L)	1 · 2–3 · 2	2 · 76	10 · 9	
Platelets (×10^9^ cells/L)	150–390	18	162	
Prothrombin time (s)	12–14	24 · 8	15 · 9	
Activated partial thromboplastin time (s)	25–37	53 · 1	67 · 3	
INR	0 · 8–1 · 5	2 · 52	1 · 43	
D-dimer (mg/L)	<0 · 3	0 · 2	0 · 2	
Urea (mmol/L)	2 · 5–7 · 5	7 · 9	14 · 8	
Creatinine (μmol/L)	44–135	161	253	
Bilirubin (μmol/L)	3 · 42–20.5	17 · 4	61 · 8	
Total protein (g/L)	60–80	30	60	
Albumin (g/L)	35–50	23	35	
Globulin (g/L)	25–35	7	25	
Alanine aminotransferase (U/L)	0–40	35 · 4	68	
Aspartate aminotransferase (U/L)	0–37	67	479 · 8	
Lactate dehydrogenase (U/L)	109–245	127	585	
Creatinine kinase (U/L)	24–195	146	9868	
C-reactive protein (mg/L)	0–10	73	150	
Glucose (mmol/L)	3 · 9–6 · 1	19	5 · 1	
Ka (mmol/L)	3 · 5–5 · 1	2 · 6	3 · 03	
Na (mmol/L)	135–147	60	114	
Cl (mmol/L)	95–106	40	80	
Ca (mmol/L)	2 · 25–2 · 75	1 · 62	2 · 09	
Urinalysis				
PH value	5 · 0–7 · 0	5 · 5	5 · 0	
Protein (mg/dl)		100	100	
Ketone (mg/dl)		15	Neg	
Bilirubin (mg/dl)		0 · 5	0 · 5	
Urobilinogen (mg/dl)		8	4	
Blood (mg/dl)		Neg	1	
Leu (leu/ul)		75	Neg	
Bacterial Diagnostic test				
Microscopy	-	+	+	+
RT-PCR	-	+	+	+
IHA	-	1:40 (serum)	1:2560 (serum) 1:160 (tissue fluid)	1:800 (serum)
rIHA	-	1:6400 (nasopharyngeal swab) 1:12800 (sputum)	1:1024 (serum)	1:5120 (serum)

Results for when the patients presented and the patients’ most abnormal result during disease progression are given. If the reading at presentation was the most abnormal reading, only one result is given

As the third patient’s condition was much threated and death in only one hour, the blood sample had not gathered and many laboratory measurements had not received

### Clinical features and outcomes of the patients

The clinical characteristics of the patients are shown in Table 1. Fever, cough, and dyspnea were the most common symptoms. haemorrhage spots can be seen all over the body at the critical period of the plague. The pulse was faster than normal (>100 b/min), while the respiratory rate was also fast. Spo2 was lower than normal. The breathing was low in the left lung and there was a moist rale in both lungs in patient 1, the breathing was low in both lung and there were moist rales in both lungs in patient 2. There were wheezing and moist rales in both lungs in patient 3.

The laboratory results of the patient 1 and 2 are shown in Table 2. As the health condition of patient 3 was very serious and he died 1.3 h after admission to the hospital, blood and urine sample had not been gathered and the laboratory measurements had not received. The Haemoglobin was increased; Prothrombin time and Activated partial thromboplastin time were prolonged. Platelets count was normal or decreased. Elevated levels of aspartate aminotransferase, creatine and urea were observed in the two patients. C-reactive protein was increased obviously. The electrolytes such as K^+^ Na^+^, Cl^−^, and Ca^2+^ were various degrees decreased. There were protein and urobilinogen in urine.

**Fig. 1:**
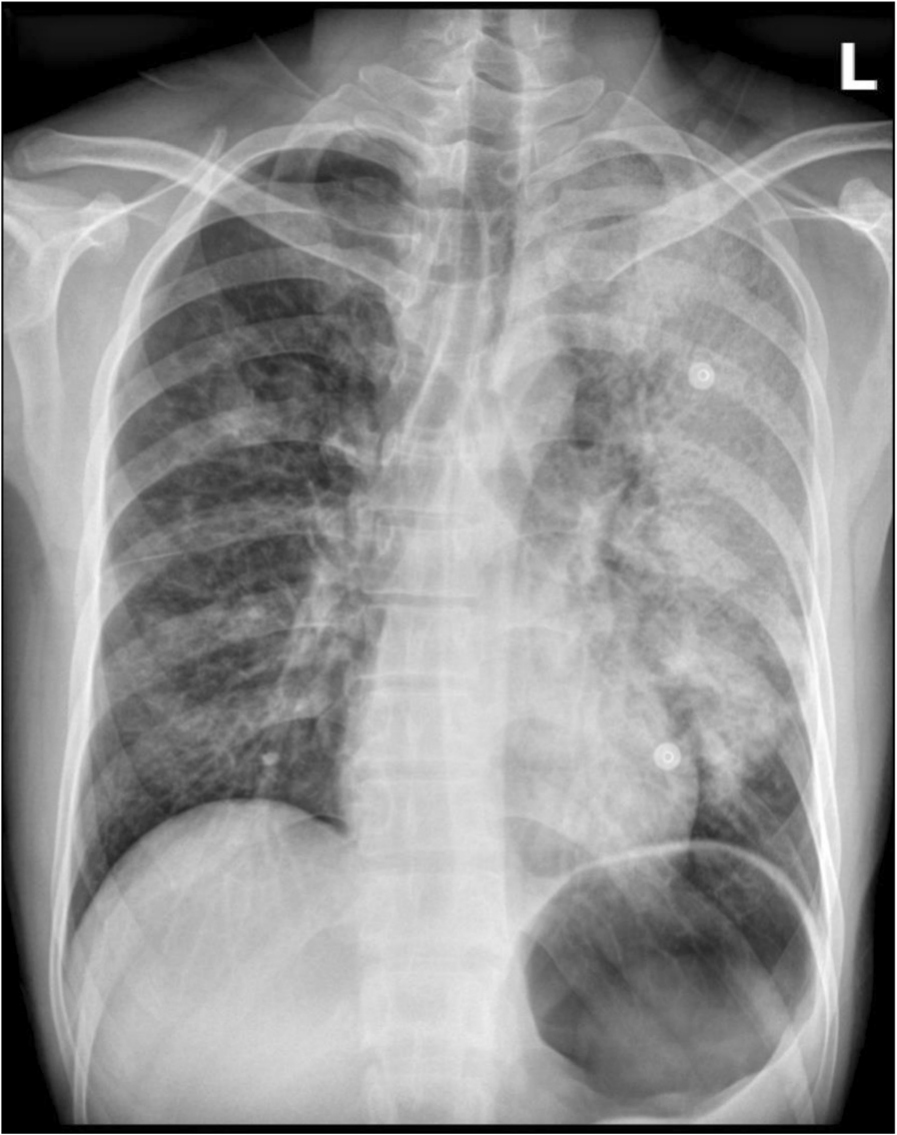
Radiographs of the chest of patient 1. Patient 1. Chest radiograph. Spotted and flocculent shadows in the upper and middle right lung field and large flakes of shadows in the left lung field were noted on July 15

**Fig. 2:**
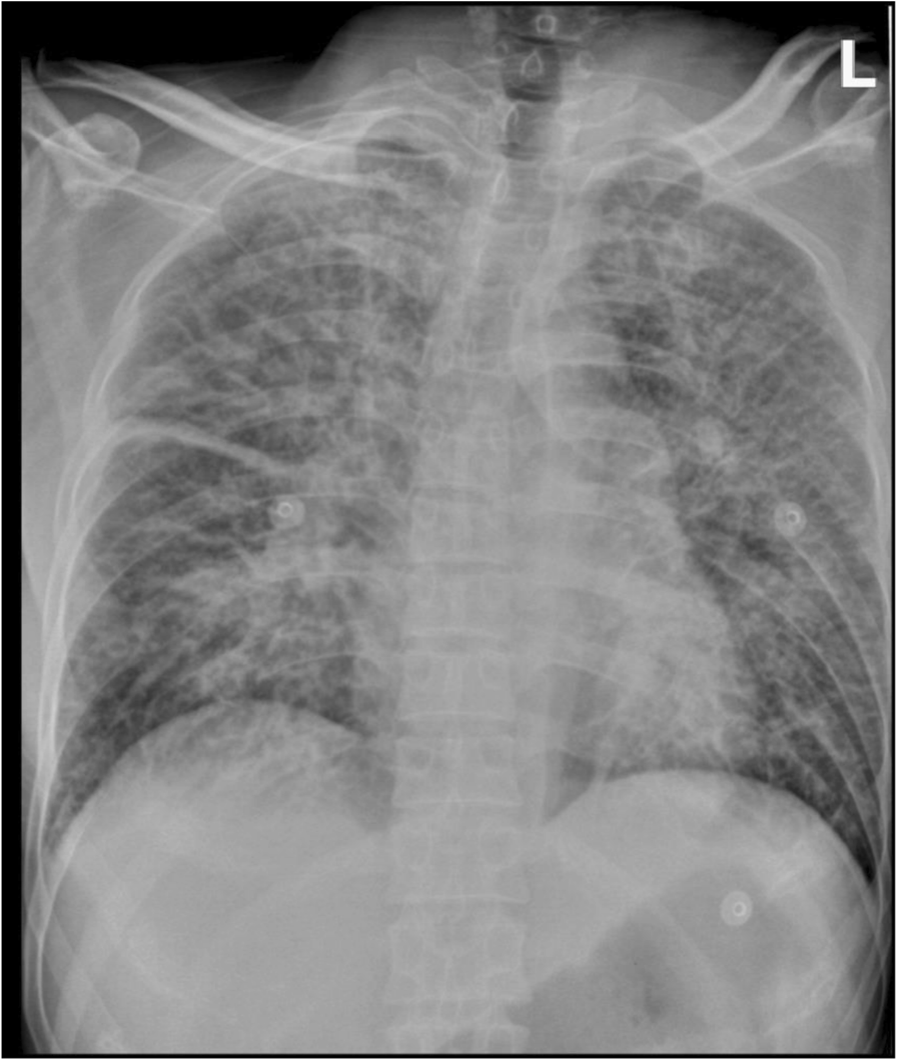
Radiographs of the chest of patient 2. Chest radiograph. Increase of pulmonary markings in both lungs, little flakes of shadows in the both lung field, especially around the hila; ribbon thickened along the interlobar pleura were noted on Oct 1

It was very valuable to take chest radiography for patient 1 and 2. Chest radiographs showed spotted and flocculent shadows in the upper and middle right lung field and large flakes of shadows in the left lung field in patient 1 on July 15. Increased pulmonary vascular markings in both lungs, little flakes of shadows in the both lung field, especially around the hila; ribbon thickened along the interlobar pleura were noted in patient 2 on Oct 1 (Figs. 1 and 2).

Several complications of the illness were observed. All the patients had ARDS. Patient 1 and 2 had septic shock, acute renal damage, acute liver injury, encephalopathy, DIC and electrolyte disturbance.

All the three patients accepted mask hydrogen and the flow rate of oxygen of patient 3 was as high as 6 L/min. Antibiotic therapy was administered in patient 1 and 3. Patient 1 received intravenous cefoperazone (1.5 g) and sulbactam (4 ml) at first, and then antibiotic were changed into streptomycin (1 g) and gentamicin (8 mg); Clindamycin (0.9 g) was administrated intravenously in patient 3.

As all the patients’ condition progressed before administration, the patients missed the treatment window. For patient 1, the local village clinic and local hospital misdiagnosed the disease because of limited medical facilities. Despite timely emergency treatment, patient 1 died 14 h after admission owing to progressive dyspnea. Patient 2 died 3 h after admission owing to Septic shock and dyspnea. Patient 3 died of refractory hypoxemia after 1.3 h in the hospital.

### Quarantine of contacts and biosafety disposal

Patient 1 had 151 contacts, patient 2 had 41 and patient 3 had 22. All the contacts were immediately were quarantined at home. IHA assay was used to detect F1 antibody in paired sera of the contacts. 3 contacts of Patient 1, 1 contact of Patient 2, and 1 contact of Patient 3 showed weak positive result. The remaining contacts were negative. Preventive medications such as streptomycin intramuscular (0.75 g) and oral sulfadiazine (2 g per day, in three doses per day), were given to all the contacts: All contacts were quarantined for 9 days under professional medical observation.

The corpse and contaminated objects were sent to a biosafety disposal unit and the area of contamination was sterilized. All dogs belonged to the patients as herding dogs that might have come into contact with the patients (five of patient 1 and three of patient 3) with F1 antibody positive were culled. Marmot depopulation was performed in the grazing location.

## Discussion

Plague, one of the most serious infectious diseases threatening human life, has strong infectivity and a high mortality rate. It can spread quickly and widely. In the Prevention and Control Act of Infectious Diseases in China, it has been listed as the first infectious disease in class A. there have been three pandemics of plague in recorded history, which has claimed numerous lives. *Y. pestis* can also be manufactured into bioterrorism weapon to threaten the world peace at present. Therefore, the prevention and control of plague is of great importance.

The bubonic or septicemic infections are usually caused by flea bite, whereas pneumonic infections are thought to be transmitted by aerosol infection from one infected individual to another and can be associated with direct contact with sick animals or ones that have recently died.

The three cases respectively occurred in Gansu Province. Gansu is located in the Qinghai–Tibet natural plague area, the largest natural plague focus in China. It witnessed annually outbreaks of plague in animals which also spread to mankind occasionally [2, 4]. The majority of human plague cases today are the result of cross-infection occurring from wild animal reservoirs, including prairie dogs, squirrels, marmots, and other small rodents or larger mammals that have become infected, such as cats and coyotes [13]. For patient 1 and 3, *Y. pestis* might have been transmitted from infected dogs, and for patient 2, it may spread from infected marmots. All those dogs are herding dogs. They were infected with *Y. pestis* without symptoms and only showed the presence of F1 antibody. There were some cases involved dog to human pneumonic plague transmission [10, 14], which provides additional evidence of the possibility of dogs as a risk of transmission of pneumonic plague to humans. It is known that domestic dogs have a relatively mild or asymptomatic infection with plague, but could be a source of human infection for the owners when their dogs are exposed to infected wildlife, thus our study adds useful evidence to support this theory. The incubation period of plague is generally two to five days, and primary pneumonic plague develops symptoms 1–2 days or even several hours after infection [15]. The incubation period of patient 1 is 2 days, and the infect time of patient 2 and 3 is unknown.

The three cases we report showed clinical features similar to influenza or other upper respiratory infections, so nobody has realized the possibility of plague and diagnose it promptly. Fever and respiratory symptoms with cough and dyspnea are the predominant clinical symptoms, with a rapid progress to deterioration of oxygenation and consciousness. Because the basic lesion of plague is vascular endothelial cell injury, acute hemorrhagic and necrotic lesions, haemorrhage spots can be found all over the body in patient 1 at the final period of the disease, at the chest and abdomen in patient 2, but not in patient 3. The breathing was low in the left lung and there was a moist rale in both lungs in patient 1, the breathing was low in both lung and there were moist rales in both lungs in patient 2. There were wheezing and moist rales in both lungs in patient 3. These symptoms were consistent with the chest radiographs. Chest radiographs showed large flakes of shadows in the left lung field in patient 1 and little flakes of shadows in the both lower lung field in patient 2.

Chest radiography demonstrations of pneumonic plague include hemorrhagic necrotizing inflammation, which may involve multiple pulmonary lobes or segments. The manifestations are mass-like lesions they may fuse into flakes and even white lung change [16]. The demonstrations tally to the pathology of lung as Doctor Wei has reported [17]: the pulmonary congestion swelling, Surface vessel hyperemia, part of the blood vessels had thrombosis. After cutting open the lung, large dark red hemic exudation overflowed. The pneumorrhagia is shown in chest radiographs as spotted and flocculent shadows, and flake-like infiltrated shadows. Laboratory parameters demonstrated the progression of the disease and the associated complications, although these parameters were not the diagnostic criteria of the plague. The Haemoglobin increased, prothrombin time and activated partial thromboplastin time was prolonged, and platelets count maintained normal or decreased. Those Laboratory parameters showed the severity of the disease. C-reactive protein increased obviously indicated the severity of the disease. As the disease progressed, many organs were involved. Elevated levels of aspartate aminotransferase showed the damage of liver, creatine and urea increased demonstrated the disorder of the renal, as well as the protein and urobilinogen in urine. The electrolytes like K^+^, Na^+^, Cl^−^ and Ca^2+^ were all decreased. The disease results in the electrolyte disturbance, and the electrolyte disturbance in turn leads to the deterioration of diseases [18].

*Y. pestis* can be detected in the laboratory through both bacteriologic and serologic methods. A variety of samples, including blood, aspirates from involved lymph nodes, skin scrapings, cerebrospinal fluid, urine, and sputum, can be used for diagnosis [19]. In the three cases, the *Y. pestis* strains were isolated from sputum, blood, lymphatic fluid, and nasopharyngeal swab samples by smear and bacterial culture. *Y. pestis* is Gram-negative oval shaped short bacillus having two blunt round ends which was darkly stained under microscopy [20]. About 50–80 % of the plague cases are positive by smear, while the positive rate of early stage bubonic plague by blood culture is 70 %, and that of advanced bubonic plague by blood culture is about 90 %. The positive rate can reach 100 % during sepsis palgue [21]. The growth of *Y. pestis* is slow; colonies are visible on plates after 48 h, and it is recommended that plates be incubated for a total of 7 days before being discarded [13, 15]. The capsule-like fraction 1 (F1) antigen expressed by *Y. pestis* is a known specific marker for identified the bacteria; therefore, the detection of F1 is important for *Y. pestis* recognition [22]. There were many highly sensitive immunological and biochemical assays to detect F1 antigen, such as the indirect hemagglutination assay (IHA, the gold standard in detection of *Y. pestis*) [23], polymerase chain reaction (PCR) analysis [24], enzyme-linked immunosorbent assays (ELISAs) [25, 26], the fiber optic biosensor measurement to fluorescence antibody staining [27, 28], and radioimmune precipitation test [29]. In China, we used IHA, RIHA, and PCR to detect F1 antigen. These techniques all have high sensitivity and specificity; however, the requirement for skilled technicians, long assay times (minimum of several hours), and the complexity of operation procedures limits their use in the field. As the key to the plague prevention and control is “early detection, early quarantine and early treatment”, what needed now is new techniques to detect the F1 antigen easily, quickly, sensitively and specifically. Because of the high mortality of plague, effective treatment is crucial. Now antibiotic treatment is the recognized worldwide effective treatment to plague. As these three patients missed the treatment window, although patient 1 and 3 had used antibiotic, all of them were dead. So using antibiotic in time is important to save the plague patients’ lives. The antibiotics include streptomycin, gentamicin, tetracycline or doxycycline and chloramphenicol were most commonly used in clinical [13, 15] and recommend by WHO [30, 31]. Fluoroquinolones, such as ciprofloxacin, are also quite effective in animal studies [32] and in vitro [33], so some experts think that fluoroquinolones is effective in the treatment of plague [20]. Some doctors also used moxifloxacin in clinical to treatment the plague in China [16]. In the above antibiotics, streptomycin was the most efficacious, which are available in clinical therapy. Plague was treated based on clinical classification, as to the pneumonic plague, the proposed starting dose of streptomycin is 2 g recommended by China CDC [34]. Unfortunately streptomycin should never be administered during pregnancy because irreversible deafness has occurred in children exposed to streptomycin in utero [35]. So this drug often is not commonlly available in the United States and some other countries, and an acceptable and preferred alternative to streptomycin is gentamicin which had been proved effective in some cases alone [36, 37]. Patient 1 had succumbed to the toxic shock before the antibiotic worked, so early diagnosis and treatment is of the most importance to improve the prognosis [30]. Patient 2 did not use any antibiotic, and patient 3 used Clindamycin which is ineffective for plague. So, if not properly treated mortality may reach high levels, particularly for pneumonic plague [38]. Recent years, several studies [39, 40] suggested that drug combination may be more effective for severe cases, for example, Streptomycin or gentamicin combined with tetracycline or doxycycline [20]. While there came out streptomycin resistant case [41], several investigators have tried to find alternatives to antibiotics treatment to pestis strains such as immunotherapy, non-pathogen-specific immunomodulatory therapy, phage therapy, bacteriocin therapy, and treatment with inhibitors of virulence factors [42].

Except etiological treatment-antibiotic therapy, other treatments were also important, which include general treatment and monitoring indicators, anti-shock treatment, respiratory support therapy, correct diffuse intravascular coagulation, maintain other important viscera function, nutrition support, and so on [34].

All contacts received prophylactic antibiotic therapy for 7-day course, as coming within 6 feet of a person with pneumonic plague before that person has received 48 h of appropriate antibiotics [7]. A range of antibiotics were administrated including tetracycline, doxycycline, ciprofloxacin, streptomycin and sulfadiazine. Meanwhile all the contacts were immediately quarantined or isolated at home for 9 days under professional medical observation. If there is a clinical suspicion of plague before laboratory confirmation, contact precautions are indicated until 2 days after the administration of antibiotic to prevent the spread of the disease [15]. Although F1 antibody of some contacts is weak positive, there was no secondary case due to receive timely prophylactic treatment.

Besides isolation and quarantine of the contacts, personal protection for medical staff, nosocomial infection control, biosafety disposal and disinfection were also important to prevent the spread of the plague. Culling the marmot and dogs in the grazing location with the patients would also help to reduce the morbidity of human plague.

A human vaccine for plague does exist, and that a vaccination strategy might be useful for individuals such as animal herders or farm workers who are occupationally exposed in areas where endemically infected reservoirs of plague-infected small mammals co-exist.

## Conclusion

Early and correct diagnosis and appropriate antibiotic treatment is essential to saving the lives of plague patients and reducing mortality. Highly alert of plague is the key to early correct diagnosis. Doctors in natural foci of plague need to improve the awareness of plague prevention. To avoid misdiagnosis, patients with clinical suspicion of plague need to be asked about the exposure history. Health education in natural plague foci should be strengthened. A vaccination strategy might be useful for individuals in natural plague foci. If people who had exposed to risk factors then develops related clinical symptoms, he/she must go to hospital without delay, so as not to miss the treatment window.

### Consent statement

Written informed consent was obtained from the next of kin of the patient for publication of this Case report and any accompanying images. A copy of the written consent is available for review by the Editor of this journal.
